# Recognition and treatment of sleep-disordered breathing: an important component of chronic disease management

**DOI:** 10.1186/s12967-017-1211-y

**Published:** 2017-05-25

**Authors:** Peter C. Farrell, Glenn Richards

**Affiliations:** 1 ResMed Science Center, c/o ResMed, 9001 Spectrum Center Blvd., San Diego, CA 92123 USA; 2 ResMed Science Center, Sydney, Australia

**Keywords:** Obstructive sleep apnea, Sleep-disordered breathing, Arterial hypertension, Chronic obstructive pulmonary disease, Continuous positive airway pressure, Noninvasive ventilation, Cardiovascular disease, Quality of life, Healthcare costs, Chronic diseases

## Abstract

Sleep-disordered breathing (SDB) is a highly prevalent condition, and is associated with many debilitating chronic diseases. The role of untreated obstructive sleep apnea (OSA) in arterial hypertension has been recognized in international guidelines. Treatment with continuous positive airway pressure (CPAP) is associated with clinically-relevant reductions in blood pressure. In heart failure (HF), SDB is associated with worse prognosis and increased mortality. Major HF guidelines recommend that patients should be treated for sleep apnea to improve their HF status. Severe OSA increases the risk of arrhythmias, including atrial fibrillation, influences risk management in stroke, and is highly prevalent in patients with type 2 diabetes. Effective treatment with CPAP improves the success of antiarrhythmic interventions, improves outcomes in stroke and reduces hyperglycemia in diabetes. Patients with coronary artery disease also have a high prevalence of SDB, which is independently associated with worse outcomes. The role of CPAP for secondary cardiovascular prevention remains to be determined. Data from large, well-conducted clinical trials have shown that noninvasive ventilation, targeted to markedly reduce hypercapnia, significantly improves survival and reduces readmission in stable hypercapnic chronic obstructive pulmonary disease. The association of SDB with chronic diseases contributes to the high healthcare costs incurred by SDB patients. SDB also has an important negative impact on quality of life, which is reversed by CPAP treatment. The high prevalence of SDB, and its association with diseases that cause significant morbidity and mortality, suggest that the diagnosis and management of SDB is an important therapeutic goal. First, adherent CPAP treatment significantly improves the quality of life of all patients with SDB; second, it eliminates the negative impact of untreated SDB on any associated chronic diseases; and third, it significantly reduces the increased costs of all hospital and medical services directly associated with untreated SDB. In short, the recognition and treatment of SDB is vital for the continued health and wellbeing of individual patients with SDB.

## Background

US data show that sleep-disordered breathing (SDB) with an apnea-hypopnea index (AHI) of ≥15/h is present in 20% of the adult population aged 30–70 years and approximately one-third of those aged between 50 and 70 years [1]. Figures from a more recent European study showed that nearly 50% of males and 23% of females in a randomly selected sample of healthy patients living in Lausanne, Switzerland had moderate to severe SDB [2]. Using the same definition, data from a systematic review reported the worldwide prevalence of SDB as ranging from 6 to 49% in different patient groups, with risk factors for obstructive sleep apnea (OSA) including increasing age, male sex and higher body mass index [3].

These statistics are alarming given that it is now well established that untreated SDB, including obstructive sleep apnea (OSA), has been linked with, and may even contribute to the development of, some of the most debilitating and costly chronic diseases. These include most forms of cardiovascular disease [4–14], cancer, chronic obstructive pulmonary disease (COPD) [15], stroke [16, 17] and type II diabetes mellitus [18, 19], conditions associated with significant morbidity and mortality in the Western world. The prevalence of SDB/OSA in these disease states varies, with rates of 3–66% in COPD [15, 20, 21] and approximately 75% in congestive heart failure (HF) and stroke [22–25]. Given that SDB is thought to contribute to the development and severity of many of these conditions [5, 17, 18, 26–29], untreated SDB/OSA is an important global public health problem.

This article provides an overview of current published evidence for the influence of SDB and PAP therapy on several important comorbidities, including incidence, disease progression, healthcare costs and quality of life.

## Pathophysiological mechanisms

The damage caused by untreated SDB/OSA is largely a result of repetitive hypoxia due to airway obstruction, concomitant activation of sympathetic neural activity (SNA), hemodynamic disturbances and sleep disruption [30–34]. Activation of the sympathetic nervous system is associated with the release of catecholamines (epinephrine and norepinephrine), which cause vasodilation (heart and brain) or vasoconstriction (all other organs) (termed the ‘fight or flight’ response) up to hundreds of times per night. The physiologic effects of OSA have been associated with systemic inflammation, with OSA patients showing increased levels of key inflammatory mediators such as tumor necrosis factor-alfa, C-reactive protein, and chemokines [35–40]. Furthermore, intermittent and chronic hypoxia has been associated with glucose intolerance and insulin resistance [41, 42], and the adverse effects of hypoxia on glucose metabolism appear to be mediated via the sympathetic nervous system [39, 43]. In addition, to effects on physiological processes, the sleep disruption associated with SDB has effects that are often very debilitating, including daytime sleepiness [44], cognitive impairment [45, 46], depression [47], relationship difficulties [48] and dementia [46] to appetite changes/obesity [49] and reduced immunity [50].

## Incidence and development of comorbidities

SDB is strongly associated with several of the most important chronic diseases affecting Western societies, including cancer, cardiovascular disease, heart failure, diabetes, stroke and COPD. In each of these diseases, the adverse physiological consequences of SDB described above contribute to accelerated disease progression.

### Arterial hypertension

A role for OSA in the pathogenesis of arterial hypertension has been widely documented and is recognized in international guidelines [26, 27]. However, many healthcare professionals remain unaware that OSA is a risk factor for arterial hypertension [51]. There is a strong bi-directional relationship between OSA and arterial hypertension, with about 30–40% of patients with arterial hypertension having clinically relevant OSA and approximately half of all patients with OSA having hypertension [12]. The prevalence of OSA is as high as 80% in patients with severe drug-resistant hypertension [52, 53], and the prevalence of arterial hypertension increases as OSA severity increases [54–61].

### Congestive heart failure

SDB is believed to play a major role in congestive HF [6, 14]. The prevalence of SDB in patients with HF with preserved ejection fraction (HF*p*EF; diastolic HF) or HF with reduced ejection fraction (HF*r*EF; systolic HF) is around 75% [23, 25], and half of these patients have moderate-to-severe sleep apnea [25]. OSA appears to be the predominant type of SDB in patients with HF*p*EF, with a prevalence of 62% compared with 18% for central sleep apnea/Cheyne Stokes respiration (CSA/CSR) [62], but prevalence rates for CSA/CSR and OSA are approximately equal in patients with HF*r*EF [63]. The severity of ventricular dysfunction increases the risk for CSA/CSR in both groups. Untreated OSA and CSA/CSR are independent risk factors for worse prognosis and death in HF patients [7–9, 13], and daytime CSR is a significant independent predictor of mortality in those with severe congestive HF [4].

### Arrhythmias

Sleep apnea is highly prevalent in patients with arrhythmias, particularly atrial fibrillation (AF). More than 50% of patients with paroxysmal or persistent AF have been shown to have clinically-relevant SDB [64], and the prevalence of SDB in patients with AF presenting for electrical cardioversion is approximately 75% [23]. Severe OSA increases the risk of AF, ventricular premature beats and non-sustained ventricular tachycardia (NSVT), and nocturnal sudden cardiac death [11]. An AHI of >20/h was a significant and independent risk factor for incident sudden cardiac death in a study of more than 10,000 patients referred for polysomnographic assessment of SDB [5].

### Coronary artery disease

The prevalence of OSA in patients with coronary artery disease (CAD) is high (up to 87% in patients referred for coronary artery bypass graft surgery) and is significantly greater than in the general population [65–70]. The prevalence of OSA in CAD is higher than that of better known risk factors such as obesity, arterial hypertension, diabetes, or AF [71], and OSA remains a major risk factor for CAD even after controlling for other risk factors, including body mass index (BMI), hypertension, hypercholesterolemia, diabetes and smoking [29]. The Sleep Heart Health Study showed that men aged 40–70 years with an AHI of ≥30/h were 68% more likely to develop CAD compared with those who had an AHI of <5/h [6]. In the recent Sleep and Stent study, OSA was a significant independent predictor of major adverse cardiac and cerebrovascular events in patients undergoing percutaneous coronary intervention (HR 1.57, 95% CI 1.10–2.24; p = 0.013) [10].

CAD patients with OSA have a worse prognosis than those without SDB. Cardiovascular event rates over 5-years’ follow-up were 37.5 and 9.3% in CAD patients with versus without OSA, respectively (p = 0.018), and untreated OSA was a significant independent predictor of cardiovascular mortality [72]. In another study in patients with acute coronary syndromes followed-up for a mean of 227 days, OSA was identified as an independent predictor of major cardiac events (hazard ratio [HR] 11.62, 95% confidence interval [CI] 2.17–62.24; p = 0.004) [73].

### Stroke

The prevalence of OSA in patients with stroke is up to 71%, based on data from case–control studies [22, 24]. An AHI of ≥20/h appears to increase the risk of having a stroke [24], and severe OSA (AHI ≥ 30/h) was significantly and independently associated with wake-up stroke in a prospective study [17]. OSA is also an independent risk factor for stroke recurrence [16].

### Diabetes mellitus

The prevalence of type 2 diabetes mellitus is increasing rapidly worldwide, largely due to the obesity epidemic, and the vascular complications of diabetes alone are a significant burden on healthcare systems [74]. SDB is highly prevalent in patients with type 2 diabetes, with up to two-thirds having at least mild OSA [75, 76]. Data from 1387 participants in the Wisconsin Sleep Cohort identified an increasing prevalence of diabetes as the severity of SDB increased; the odds ratio for having diabetes in patients with an AHI of ≥15/h versus <5/h (adjusted for age, sex and BMI) was 2.30 (95% CI 1.28, 4.11; p = 0.005) [19]. The Sleep Heart Health Study was one of the largest community-based cohort studies utilizing polysomnography (PSG), and the results indicated that OSA was associated with impaired fasting glucose, glucose intolerance and type 2 diabetes mellitus, even when accounting for common confounding factors such as age, sex, race, waist circumference and BMI [18].

### Chronic obstructive pulmonary disease

Although less common in the general population (1–4%), the coexistence of COPD and OSA (termed ‘overlap syndrome’) is relatively common in patients with either disease (3–66%) [15]. Sleep, via reduced chemosensitivity, hypotonia and supine posture, places an additional load on breathing compared with periods of wakefulness, and patients with COPD often have poor sleep quality [77]. The presence of OSA in this setting further increases breathing load and sleep disruption, and adds to the cardiovascular effects of repeated apneas. Potential mechanisms by which OSA could have a negative influence on COPD include vagally-mediated bronchoconstriction (thus augmenting expiratory airflow resistance) and increasing resistive load in the lower airways due to high negative intrathoracic pressure [78–80]. Patients with both COPD and OSA have high rates of morbidity and mortality, and poor quality of life [80–86]. In addition, the coexistence of OSA and COPD is associated with high healthcare utilisation and costs [87].

### Cancer

The connection between SDB and cancer is a very interesting one and is the direct result of repetitive hypoxia. If cancer cells are exposed to repetitive hypoxia in vitro, almost instantaneous angiogenesis occurs, with proliferation of blood vessels and cancer cells [88]. In animal models of cancer, intermittent hypoxia has been linked with increased tumor cell proliferation and metastases [89, 90]. From a clinical perspective, a 22-year prospective study involving over 1500 subjects showed that all-cause cancer mortality was increased nearly fivefold in patients with severe OSA (apnea-hypopnea index [AHI] > 30/h), while those with moderate-to-severe OSA (AHI 15–30/h) had a doubling of all-cause mortality [91]. In an overview of current evidence from human studies, it was concluded that study findings are largely convergent, suggesting an association between OSA and cancer, and that further research is warranted [28].

## Effects of treatment

SDB/OSA can be effectively and safety treated using positive airway pressure (PAP) therapies. PAP is delivered nasally, using either a nasal mask or prongs, or a full face mask. Millions of patients worldwide already receive this therapy, which is typically delivered at a pressure of approximately 10 cm H_2_O (or about 1% of atmospheric pressure). Furthermore, screening for SDB, its definitive diagnosis, and initiation of treatment can be cost effective and easy using home-based diagnosis and automated CPAP treatment, with optimal mask fitting done by a trained respiratory technician or nurse [92–94]. Since its introduction 25 years ago, CPAP has changed lives for the better in over 10 million patients worldwide.

There are few published randomised trials on the effects of treatment on disease progression because ethical considerations preclude randomization of OSA patients to no therapy for long periods in a clinical trial setting, but there are many observational studies demonstrating the benefits of CPAP therapy. Therefore, there is a considerable and growing body of evidence suggesting that effective treatment of SDB/OSA with CPAP also attenuates some of the negative consequences of untreated sleep apnea and the rate of progression of many comorbid conditions [34, 133–135, 137, 95–104]. Furthermore, it has been shown that patients compliant with PAP treatment are much less likely to use inpatient and outpatient services, resulting in substantial cost savings for the healthcare system [105–110].

### Arterial hypertension

In the most recent meta-analysis of published data, which included 32 randomized controlled trials, mean reductions in daytime systolic and diastolic blood pressure (BP) with CPAP versus no treatment were 2.6 and 2.0 mmHg, respectively; corresponding night-time reductions were 3.8 and 1.8 mmHg (p < 0.001) [111]. While these effects are modest, most patients in these studies had well-controlled hypertension before CPAP was initiated. Therefore, BP reductions during CPAP were additional to those associated with antihypertensive drug therapy, and were of a magnitude that was at least half of that achieved with drug therapy alone in similar analyses. The meta-analysis also showed a link between baseline OSA severity and the beneficial effects of CPAP on BP, with the greatest reductions seen in those with more severe OSA (β ± standard error, 0.08 ± 0.04) [111]. The most substantial reductions in BP during CPAP therapy have been seen in patients with drug-resistant hypertension [53]. A meta-analysis of five randomized clinical trials of CPAP in patients with OSA and resistant hypertension reported pooled reductions of 4.78 and 2.95 mmHg in 24-h ambulatory systolic and diastolic BP, respectively [100].

Based on combined data from three randomized trials, increases in mean office and home systolic BP after CPAP was stopped were 5.4 and 9.0 mmHg, respectively (p < 0.003 and p < 0.001 vs on CPAP, respectively). Corresponding increases in mean diastolic BP were 5.0 and 7.8 mmHg (both p < 0.001). The changes were described as clinically relevant by authors of the analysis [112]. In addition, there was a significant association between OSA severity and the extent of BP increase in response to CPAP withdrawal [112].

### Congestive heart failure

Two randomized trials evaluating the effect of CPAP therapy in patients with OSA and HF showed significant improvements in left ventricular ejection fraction (LVEF) after 1 and 3 months and improved quality of life [98, 101]. A Japanese cohort study documented a positive effect of CPAP treatment on survival in HF patients with OSA, and also highlighted the importance of compliance with therapy in achieving beneficial outcomes [99]. Treating OSA in patients with HF*p*EF has been shown to improve left ventricular diastolic function [95]. The 2010 Heart Failure Society of America Comprehensive Heart Failure guidelines [113] and the 2013 American College of Cardiology Foundation (ACCF)/American Heart Association (AHA) guidelines [114] recommend that HF patients with OSA should receive treatment for sleep apnea to improve their HF status.

CPAP has limited ability to treat CSA/CSR in patients with HF [115–118], and the Canadian Positive Airway Pressure (CANPAP) study, which investigated the effect of CPAP therapy on CSA/CSR on transplantation-free survival in patients with stable HF, did not show a positive effect of therapy on either transplant-free survival or hospitalization [119]. Adaptive servo-ventilation (ASV) is a PAP therapy designed specifically to treat CSA/CSR, and is the most effective treatment for this type of SDB in HF patients [120–123].

Smaller trials of ASV have documented improvements in AHI, sleep quality, quality of life, LVEF, New York Heart Association class, oxygen uptake, natriuretic peptide levels, inflammatory markers and exercise capacity [124–127], with these results being consolidated in a recent meta-analysis [128]. The effect of ASV treatment on morbidity and mortality was investigated in the SERVE-HF study (NCT00733343) [129], which enrolled 1325 patients with chronic stable HF*r*EF and CSA/CSR, and finished in mid-2015 [130]. The effects of using ASV in HF*r*EF patients overall was disappointing, and patients with the most severe disease (LVEF <30 and >50% CSR) randomized to ASV had a higher mortality rate than those in the control group [130]. Conversely, patients with an LVEF ≥ 30% appeared to fare better when randomized to ASV [130]. On the basis of these results, ASV is contraindicated in patients with HF*r*EF and LVEF ≤ 45%. However, ASV still has a role in a number of other indications, including HF*p*EF.

The cardiovascular improvements with minute ventilation-targeted ASV therapy in heart failure (CAT-HF) study (NCT01953874) compared the effects of targeted ASV added to optimized medical therapy compared with medical therapy alone in patients with acute decompensated HF [131]. The primary endpoint (a global rank score based on a hierarchy of death, cardiovascular hospitalizations and percent changes in 6-min walk distance at 6 months) did not differ significantly between the ASV and control groups (p = 0.92), and there was no significant interaction between treatment and LVEF. However, a subgroup analysis suggested that ASV may improve outcomes in patients with HF*p*EF, and sleep apnea parameters were significantly improved by ASV in all patients [131].

### Arrhythmias

The effectiveness of antiarrhythmic drugs and ablation interventions for the treatment of AF is reduced in patients with severe OSA [132]. Treatment of OSA with CPAP significantly reduces the risk of AF recurrence after ablation therapy [133], with one small study finding that the 1-year arrhythmia recurrence rate after successful cardioversion was halved when OSA was treated with CPAP [134]. In a larger study (n = 426), CPAP therapy was associated with a higher AF-free survival rate (71.9 vs 36.7% in untreated patients; p = 0.01) in patients with OSA and AF undergoing pulmonary vein isolation. The proportion of patients who were free of AF without drug treatment or repeat ablation was also significantly higher in CPAP users versus non-users [135]. Co-existing HF and SDB increases the risk of developing malignant ventricular arrhythmias [136], but this can be reduced by treating CSR with ASV [137]. Effective treatment of OSA is recommended by the electrophysiological societies of Europe and North America [138].

### Coronary artery disease

Treatment of OSA with CPAP has been shown to have a beneficial effect on cardiovascular event rates and mortality in patients with coronary artery disease. In one study the risk of fatal and nonfatal cardiovascular events in OSA patients treated with CPAP was 36% lower than in untreated patients, a finding that has been repeated in studies with longer follow-up durations [96, 97, 102, 104]. However, the results of the international, multicenter sleep apnea cardiovascular endpoints (SAVE) study, conducted in non-sleepy patients with OSA and existing coronary or cerebrovascular disease, were neutral [139]. The rate of the primary endpoint (a composite of death from cardiovascular causes, myocardial infarction, stroke, or hospitalization for unstable angina, heart failure or transient ischemic attack) was similar in patients treated with CPAP + usual care and usual care alone (HR 1.10, 95% CI 0.91–1.32; p = 0.34). Thus, the addition of CPAP to usual care did not reduce cardiovascular events when used as a secondary prevention intervention in OSA patients with coronary or cerebrovascular disease in this study. However, average CPAP usage was only 3.3 h/night, a level most clinicians would define as inadequate treatment. Even with usage levels this low, the CPAP group showed significantly improved daytime alertness, mood, and quality of life, and the number of self-reported work days lost due to ill-health was reduced. This is the first large sleep apnea study demonstrate that treatment with CPAP has a positive impact on depressive symptoms.

Ongoing studies (ISAACC [140] and TEAM-ASV) will determine the role of early PAP therapy after acute coronary syndrome, based on documented associations between SDB and infarct size/myocardial salvage [141] and right heart enlargement [142] after acute myocardial infarction.

### Stroke

Use of CPAP in patients with stroke and OSA has been associated with improvements in subjective well-being, significant reductions in the AHI, and normalization of nocturnal BP and oxygen saturation [143–145]. A reduction in 5-year mortality has been documented in stroke patients with an AHI of >20/h who tolerated CPAP compared with those who were untreated [103]. The results of these studies show that there is an increasing body of evidence that OSA plays a significant role in the overall risk management of stroke patients and that CPAP therapy can improve outcomes.

### Diabetes mellitus

Treatment of OSA with CPAP has the potential to lower fasting plasma glucose, post-prandial glucose and glycosylated hemoglobin levels to at least the same extent as oral hypoglycemic agents in patients with diabetes [34]. Other beneficial effects of CPAP therapy in these patients include reductions in blood pressure, sympathetic nerve activity and arterial stiffness [146], as well as improved quality of life [147]. Reductions in cardiovascular risk seen in cohorts of OSA patients treated with CPAP would also be expected to be seen in those with diabetes. Screening and treatment of SDB is becoming incorporated in some care pathways for diabetes [148, 149], but better recognition of SDB as an important comorbidity by endocrinologists is needed.

### Chronic obstructive pulmonary disease

Treatment of OSA with CPAP in patients with COPD has been shown to have a number of beneficial effects. Most importantly, use of CPAP decreased mortality in overlap syndrome patients [83, 150, 151], with greater reductions seen as device usage time increased [151]. The rate of COPD exacerbations can also be reduced by CPAP treatment of OSA [83].

Noninvasive ventilation (NIV) is the standard of care for treating severe acute exacerbations of COPD and is widely used. However, until recently, there was no positive study for the effect of NIV on long-term survival in chronic stable COPD patients. A landmark trial recently showed that stable hypercapnic COPD patients treated with NIV had a two-thirds reduction in mortality and improved quality of life compared to those not receiving NIV [152]. The key point in this study was that NIV was targeted to markedly reduce hypercapnia. The benefits of NIV in patients with hypercapnic COPD were reinforced by the results of the HOT-HMV study, conducted in COPD patients with persistent hypercapnia at 2–4 weeks after an acute exacerbation [153]. This randomized, controlled trial showed that the addition of home mechanical ventilation (HMV [NIV]) to home oxygen therapy (HOT) reduced the likelihood of readmission or death by almost 50%. In addition, the time to readmission or death was increased by >90 days. Overall, treatment of 6 patients with HMV was required to prevent 1 readmission or death. These results are consistent with the effects of NIV on other chronic diseases causing respiratory failure, and are expected to lead to significantly increased chronic use of NIV in COPD.

## Healthcare costs

Several studies have demonstrated that SDB is associated with higher healthcare costs, and a consistent pattern of healthcare utilization has emerged [105, 106, 109, 154–158]. OSA patients have higher direct medical costs for up to 10 years before diagnosis compared with those who are not diagnosed with OSA [105, 154–156], and the magnitude of difference in these costs is directly related to the severity of SDB [154]. Over this period, OSA patients develop comorbid disease, causing healthcare costs to increase rapidly, particularly in the few years before diagnosis [157]. By the time a diagnosis of OSA is made, a range of comorbid diseases (including arterial hypertension, type 2 diabetes, cardiovascular disease and/or HF) are usually established and healthcare costs are approximately double those in patients without OSA [105, 157].

Treatment with CPAP is most costly in the first year of therapy and direct medical costs may increase temporarily due to the cost of the device, but costs decline (or at least stop increasing at the rate before diagnosis) over subsequent years [105–110]. Established comorbidities mean that costs do not return to the levels seen in those without OSA, but costs are much lower in treated OSA patients compared to OSA patients who are not on treatment. The latter cohort have very poor outcomes and incur rapidly increasing costs as a result of advancing comorbid disease. CPAP therapy slows the rate of spending on comorbid conditions, suggesting that it does have a positive effect on disease progression.

The indirect costs of untreated OSA are higher than those for many other chronic diseases. When indirect costs are included, the societal costs of OSA are equivalent to those of stroke or congestive HF. In 2010, it was estimated that the annual cost of SDB/OSA in the US was $US67–165 billion [159]. This is, at least in part, because OSA primarily affects a working age population and impacts on performance at work. Some effects, such as presenteeism, are difficult to measure, but the combined costs of workplace and traffic accidents, and reduced time at work due to illness mean that the indirect costs of OSA exceed direct medical costs [160, 161]. These high indirect costs mean that CPAP therapy is particularly cost effective from a societal or self-insured employer perspective, and dominance over other strategies (better outcomes at lower cost) is achieved within a reasonable timeframe [162].

Recent cost utility studies show that CPAP becomes increasingly cost effective from year 2 of therapy onwards and is significantly below accepted thresholds for affordability by year 5 [162–167]. CPAP therapy is more cost effective than many of the preventative and treatment strategies currently used for cardiovascular diseases. It has similar cost effectiveness to aspirin for the prevention of cardiovascular disease in at-risk men [168, 169], is more cost effective than statins [170], and is considerably more cost effective than both cardiac resychronization therapy in HF [171] or catheter ablation of AF [172]. It is possible that initiating effective treatment for SDB earlier in the course of comorbid diseases would increase the cost effectiveness of therapy.

## Quality of life

Poor sleep quality in OSA patients is associated with symptoms such as daytime sleepiness, tiredness, lack of concentration, memory impairment, and psychological disturbances, all of which reduce quality of life [173]. Patients with OSA have higher rates of unemployment, lower incomes, earlier retirement and higher rates of divorce than those without OSA [109]. Treatment with CPAP rapidly and reliably improves sleep architecture, reduces symptoms and improves quality of life [109, 174].

Patients with the worst symptoms arguably have the most to gain from PAP therapy, but those with mild disease and minimal symptoms can also show significant improvements during CPAP therapy, as shown in a UK study in which subjects who had proven OSA but insufficient symptoms (as judged by themselves and their physician) to justify treatment were randomized to CPAP or standard care [175]. Patients treated with CPAP experienced clinically significant improvements in both sleepiness and quality of life. These results suggest that clinical assessment of patients with OSA does not reliably identify all those likely to benefit from therapy, and that all patients with OSA, not just those who complain of symptoms, should be offered and can benefit from CPAP treatment.

## Conclusion

The high prevalence of SDB, and its direct connection with many diseases which cause significant morbidity and mortality in Western societies, along with the documented ability of positive airway pressure to improve quality of life, prevent or slow progression of a wide range of common chronic diseases, and reduce healthcare resource use and costs suggest that the recognition and treatment of SDB could be described as the ‘holy grail’ of healthcare. Currently available data provide an excellent rationale for diagnosing and treating SDB/OSA, and for the inclusion of SDB screening and treatment in disease management pathways for the chronic diseases with which it is often associated. SDB/OSA can be easily and effectively treated with CPAP that, as has been stated, improves quality of life, slows and may even reverse the progression of associated chronic diseases, and significantly reduces both hospital and medical healthcare costs. In short, the screening, recognition and treatment of SDB should be a central component of chronic disease management programs.

## Abbreviations

AF: atrial fibrillation
AHI: apnea-hypopnea index
ASV: adaptive servo-ventilation
BMI: body mass index
BP: blood pressure
CAD: coronary artery disease
COPD: chronic obstructive pulmonary disease
CPAP: continuous positive airway pressure
CSA: central sleep apnea
CSR: Cheyne–Stokes respiration
HF: heart failure
HF*p*EF: heart failure with preserved ejection fraction
HF*r*EF: heart failure with reduced ejection fraction
LVEF: left ventricular ejection fraction
NIV: noninvasive ventilation
NSVT: non-sustained ventricular tachycardia
OSA: obstructive sleep apnea
PAP: positive airway pressure
SDB: sleep-disordered breathing
SNA: sympathetic neural activity
